# Real-life vaccination coverage in Slovak children with rheumatic diseases

**DOI:** 10.3389/fped.2022.956136

**Published:** 2022-08-12

**Authors:** Barbora Balažiová, Zuzana Kuková, Daša Mišíková, Katarína Novosedlíková, Tomáš Dallos

**Affiliations:** Department of Pediatrics, National Institute for Children's Diseases, Comenius University Medical School in Bratislava, Bratislava, Slovakia

**Keywords:** vaccination, rheumatic disease, children, immunosuppressive therapy (IST), recommendations

## Abstract

**Background:**

Evidence-based recommendations for vaccination of patients with pediatric rheumatic diseases (PRDs) are available, their implementation in practice is unknown.

**Objectives:**

To analyze real-life vaccination coverage in children with PRDs and identify reasons for incomplete vaccination.

**Methods:**

Up-to-date information on vaccination status of Slovak children followed at a tertiary pediatric rheumatology center was retrieved from pediatricians over an 18-month period and compared to the standard Slovak Immunization Schedule. Reasons for missed vaccinations were analyzed retrospectively.

**Results:**

Vaccination records of 156 patients (median age 10 years, 2–18) with PRDs (JIA *n* = 108, systemic diseases *n* = 21, autoinflammatory diseases *n* = 16, uveitis *n* = 9, others *n* = 2) were available for analysis. 117 (75.0%) were completely vaccinated, 2 (1.3%) had not received any vaccine due to reasons unrelated to PRD. 37 (23.7%) remaining patients missed altogether 48 mandatory vaccinations. In 58.3% (*n* = 28, in 24 patients) no PRD related reasons for missing vaccinations were identified. Only 20 vaccinations (18 live-attenuated and 2 non-live in 19 patients) were missed due to ongoing immunosuppressive treatment or PRD activity. Patients aged 11–14 years were more likely to be incompletely vaccinated than other age groups (48.8% vs. 15.9%, *p* < 0.001), mainly due to missed MMR booster. Systemic immunosuppressive treatment was a significant predictor for incomplete vaccination status (OR 5.03, 95% CI 1.13–22.31, *p* = 0.03).

**Conclusion:**

Full vaccination is possible in a high proportion of PRD patients. In addition to immunosuppressive therapy, reasons unrelated to PRDs are a frequent and possibly inadequate cause of missed vaccinations. Periodic vaccination status assessments are needed in pediatric rheumatology care.

## Introduction

Children with pediatric rheumatic diseases (PRDs) are at increased risk for more frequent and serious infections, considerably contributing to their increased morbidity and mortality (1–4). This risk may be primarily conferred by the dysregulation of the immune system inherent to the PRD itself, as documented by the increased baseline risk for serious infections reported in patients with juvenile idiopathic arthritis (JIA) even without immunosuppressive treatment (1). Additionally, disease activity has been identified as an independent risk factor for infections (5).

Systemic immunosuppressive therapy (IST) may increase susceptibility to infections: data from the Pharmachild Registry indicate that both, methotrexate (MTX) and biologics including TNF-inhibitors (TNFi), increase the risk of infection in JIA patients (6). Moreover, according to data from the German Biologics in Pediatric Rheumatology Registry, TNFi confer an increased risk for serious infections as compared to patients treated with MTX alone (5). Available data uniformly indicate a considerably increased risk for infections in patients treated with corticosteroids added to DMARDs (1, 5, 6).

Most infections in children treated for PRDs are common childhood infections of usual severity (3). However, due to IST, an increased proportion of infections may be more severe, may present with masked symptoms, or may be caused by opportunistic microorganisms (3, 7). Severe bacterial infections requiring inpatient care in children with JIA and juvenile systemic lupus erythematosus (jSLE) include mainly pneumonia, bacteremia, urinary tract infection and cellulitis (1, 2). Among opportunistic infections, herpetic infections (mostly herpes zoster) are most prevalent, followed by tuberculosis (8).

Vaccination is an effective public health measure, preventing common, potentially life-threatening infectious diseases in otherwise healthy children (9). Considering the increased risk of infections in children with PRDs, especially those with ongoing IST, vaccination with available vaccines may play an important role in their management. Despite potential benefits, some available studies indicate that the rate of fully vaccinated children with PRDs is insufficient (10–14). In spite of growing evidence for vaccine safety and efficacy in this group of children (15, 16), some questions and concerns remain, both, among physicians and parents. These include concerns about disease flare induction and vaccination-related side effects, as well as possible blunting of the post-vaccination immune response by ongoing IST (10, 17). Until recently, due to concerns about potential infection with vaccination strains, contraindication of live-attenuated vaccines during high-dose IST was considered standard of care (18). In the light of currently available evidence, vaccinations with live-attenuated vaccines can be considered with caution under special circumstances even during low-dose immunosuppression (19, 20), with emerging data supporting this approach also in children treated for PRDs, including treatment with biologics (21, 22).

Evidence-based recommendations for the vaccination of patients with PRDs are available (19, 23). However, their degree of implementation into practice remains suboptimal or even unknown (10–14). The aim of our study was to analyze real-life vaccination coverage in children with PRDs, to identify reasons for incomplete vaccination and to propose targeted actions for improvement.

## Materials and methods

### Study design

Cross-sectional analysis of vaccination coverage with mandatory and optional vaccines (with respect to the standard Slovak Immunization Schedule) and retrospective analysis of reasons for missed and delayed vaccinations.

### Patients and methods

Over a period of 18 months (January 2019 – June 2020) caregivers of children with PRDs (aged 0–19 years) followed at a tertiary pediatric rheumatology center (National Institute for Children's Diseases) were asked to provide up-to-date information on the vaccination status of their child. Information on vaccinations was retrieved from the children's pediatricians (primary care providers) and updated to the reference date (30^th^ June 2020) by contacting parents or physicians by e-mail or phone. Patients with incomplete information on the type of administered vaccines were excluded from this study, as were patients without updated vaccination status. Demographic and medical data were obtained from electronic medical records.

Vaccination status for each birth cohort was individually compared to the standard Slovak Immunization Schedule. Mandatory vaccinations in Slovakia at the time of analysis included non-live vaccines against diphtheria, tetanus, pertussis, poliomyelitis (inactivated polio vaccine, IPV), *Haemophilus influenzae type b*, hepatitis B and *Streptococcus pneumoniae* and live-attenuated vaccines against measles, mumps, rubella (MMR). Regular vaccination against tuberculosis at the age of 11 was stopped in 2010, and in 2012 for new-borns. Therefore, vaccination coverage against tuberculosis was not evaluated in our study. Vaccination coverage against pneumococci was analyzed with respect to the introduction of compulsory pneumococcal vaccination in Slovakia in 2009.

In terms of missed and delayed vaccinations, possible reasons related to the patient's PRD (disease activity and ongoing IST) were analyzed individually for each patient and vaccination with respect to the standard time frame of the Slovak Immunization Schedule and at the reference date.

The study was approved by the institutional Ethics Committee of the National Institute for Children's Diseases.

### Statistical analysis

Patients' characteristics and vaccination coverage were summarized using descriptive statistics. Fischer's exact test was used to compare the frequency between selected groups. A *p* value < 0.05 was considered statistically significant. Logistic regression was used to identify factors independently associated with incomplete vaccination status. Only significant variables (*p* < 0.05) identified in univariate analysis were included in multivariate analysis. Statistical analyses were performed using NCSS 11 Statistical Software (2016, NCSS, LLC., Kaysville, Utah, United States, https://ncss.com/software/ncss).

## Results

### Patients' characteristics

Information on the vaccination status could be retrieved from 166 out of 297 patients followed at our pediatric rheumatology center (55.9%). After exclusion of 10 patients due to unavailability of complete information, the study population consisted of 156 children with PRDs. Median age was 10 years (range: 2.5–18 years) with median disease onset at the age of 5 years (range: 0.5–16 years).

The majority of patients were diagnosed with JIA (*n* = 108, 69.2%), with the most common subtype being oligoarticular JIA (*n* = 65, 60.2% of JIA), followed by polyarticular seronegative JIA (*n* = 22, 20.4%), enthesitis-related arthritis (*n* = 9, 8.3%), systemic JIA (*n* = 7, 6.5%), psoriatic JIA (*n* = 4, 3.7%) and undifferentiated JIA (*n* = 1, 0.9%). 21 patients were followed for systemic connective tissue diseases (13.5%) (juvenile dermatomyositis *n* = 7, jSLE *n* = 5, Sjögren's syndrome *n* = 4, localized scleroderma *n* = 3, Takayasu's arteritis *n* = 1, cutaneous vasculitis *n* = 1). 16 patients (10.3%) were followed for an autoinflammatory disease (chronic non-bacterial osteomyelitis *n* = 8, PFAPA syndrome *n* = 7, Familial Mediterranean fever *n* = 1) and 9 patients (5.8%) for primary isolated uveitis. Other diagnoses included secondary macrophage activation syndrome and tendovaginitis ligamenti carpi ulnaris (*n* = 1 each) (Table 1).

**Table 1:** Patients' characteristics and treatment modalities during rheumatological follow-up.

**Variable**		
Patients with PRD / *N*		156
Females / *N* (%)		106 (67.9)
Age at the reference date—median (range) in years		10 (2.5−18)
Age at PRD onset—median (range) in years		5 (0.5−16)
**Therapy**	csDMARDs^*^ / *N* (%)	124 (79.5)
	systemic glucocorticoids (GC) / *N* (%)	47 (30.1)
	bDMARDs ^¥^ / *N* (%)	26 (16.7)
	high-dose IVIg / *N* (%)	3 (1.9)
	NSAIDs / *N* (%)	108 (69.2)
	intraarticular corticosteroids^‡^ / *N* (%)	64 (41.0)
	topical corticoids in uveitis^¤^ / *N* (%)	21 (13.5)

csDMARDs, conventional synthetic disease-modifying antirheumatic drugs; bDMARDs, biologic disease-modifying antirheumatic drugs; IVIg, intravenous immunoglobulins; NSAIDs, non-steroidal anti-inflammatory drugs.

* Including methotrexate, hydroxychloroquine, cyclosporine A, azathioprine, cyclophosphamide, sulfasalazine, mycophenolate mofetil and colchicine.

¥ Including etanercept, adalimumab, anakinra, tocilizumab and rituximab.

‡ In 43 JIA pt. used only at disease onset (n = 30 later required systemic IST, in n = 13 no other treatment modality was needed). In n = 13 only used to treat JIA relapse. In n = 8 used as initial and also relapse treatment.

¤ Including patients with primary uveitis and JIA-associated uveitis.

130 patients (83.3%) required systemic IST at any timepoint, at the reference date the number of patients receiving IST decreased to 91 (58.3%). The most commonly used drugs were conventional synthetic DMARDs (*n* = 124, 79.5%), predominantly methotrexate (*n* = 110, 70.5%). Systemic glucocorticoids, including short-time bridging therapy, were required in 47 (30.1%) children (especially in patients with systemic disease, uveitis and episodically in PFAPA syndrome), but at the reference date, corticosteroids were regularly taken only by 6 patients. Biologic DMARDs (bDMARDs) were being administered to 26 (16.7%) individuals. 56 patients (35.9%) required a combination of systemic IST (Table 1).

### Vaccine coverage with mandatory vaccines

Complete vaccination status was found in 117 of 156 patients with PRDs (75.0%). 2 children (1.3%) had not received any vaccine due to reasons unrelated to their PRD (personal beliefs of parents or unstable epilepsy). 37 remaining patients (23.7%) missed altogether 48 mandatory vaccinations (27 non-live and 21 live-attenuated).

Retrospective analysis of medical records of incompletely vaccinated patients (followed every 3–6 months) did not identify any additional long-term vaccination barriers (relevant chronic diseases, prolonged infections, prolonged changes in overall health status etc.,) other than the patient's PRD.

Vaccination status differed according to age groups. Patients aged 11–14 years were more likely to be incompletely vaccinated than other age groups (48.8% vs. 15.9%, *p* < 0.001). Complete vaccination status differed significantly between the groups of rheumatic diseases (*p* = 0.04), with incomplete vaccination status being most prevalent in systemic connective tissue disease and uveitis patients. Gender and use of optional vaccinations were not significantly associated with being completely vaccinated (*p* = 0.17 and *p* = 0.13, *respectively*), neither were age at disease onset and disease duration (*p* = 0.44, *p* = 0.14, *respectively*). Any systemic IST (OR 5.03, 95% CI 1.13–22.31, *p* = 0.03), glucocorticoids (OR 3.02, 95% CI 1.42–6.44, *p* = 0.005) and bDMARDs (OR 4.00, 95% CI 1.66–9.65, *p* = 0.003) were all associated with incomplete vaccination status (Table 2).

**Table 2:** Associations of selected variables with vaccination status.

**Variable**	**Characteristic**	***N***	**Vaccination status**		*p*-value^*^
			**Complete *N* (%)**	**Incomplete *N* (%)**	
Age groups	2–6 years	36	33 (91.7)	3 (8.3)	**<0.001**
	7–10 years	44	35 (79.5)	9 (20.5)	
	11–14 years	43	22 (51.2)	21 (48.8)	
	15–18 years	33	27 (81.8)	6 (18.2)	
Type of PRD	JIA	108	83 (76.9)	25 (23.2)	**0.04**
	Systemic CTD	21	12 (57.1)	9 (42.9)	
	Autoinflammatory diseases	16	15 (93.8)	1 (6.3)	
	Uveitis	9	5 (55.6)	4 (44.4)	
	Others	2	2 (100.0)	0 (0.0)	
Gender	Male	50	34 (68.0)	16 (32.0)	0.17
	Female	106	83 (78.3)	23 (21.7)	
Use of optional vaccinations	Yes	61	50 (82.0)	11 (18.0)	0.13
	No	95	67 (70.5)	28 (29.5)	
Use of systemic IST	Yes	130	93 (71.5)	37 (28.5)	**0.03**
	No	26	24 (92.3)	2 (7.7)	

PRD, pediatric rheumatic disease; JIA, juvenile idiopathic arthritis; CTD, connective tissue disease; IST, immunosuppressive therapy.

* Fischer's exact test. Significant values (p < 0.05) marked in bold.

In a multivariate analysis, only being aged 11–14 years (OR 4.45, 95% CI 1.92–10.34, *p* < 0.001) and taking bDMARDs (OR 3.44, 95% CI 1.29–9.20, *p* = 0.01) were independently associated with incomplete vaccination status in our cohort, as opposed to type of diagnosis, glucocorticoid and systemic immunosuppressive treatment.

### Reasons for missed mandatory vaccinations

Recent disease onset, high-dose glucocorticoid therapy and rituximab were reasons for not administering 2 of 27 missed non-live mandatory vaccinations. For the remaining 25 missed non-live vaccinations, analysis of medical records identified no PRD-related restrictions according to current recommendations, as disease remission without treatment (*n* = 2) or inactive disease or remission on standard doses of IST was achieved within the standard vaccination schedule (*n* = 13) or at the reference date (*n* = 6). Another 4 children had missed vaccinations with non-live vaccines within the standard vaccination schedule for unknown reasons before the onset of PRD. All of them could have been vaccinated at the reference date with respect to PRD and its treatment (remission without IST *n* = 2, remission with low-dose IST *n* = 1, episodic glucocorticoids for PFAPA syndrome *n* = 1).

18 of the 21 missed live-attenuated mandatory vaccinations were not administered due to ongoing IST. In 3 cases, no PRD-related restrictions were identified, as these patients were in disease remission without systemic treatment at the reference date. PRD-related restrictions in missed vaccinations are summarized in Table 3.

**Table 3:** Missed and delayed vaccinations and PRD-related restrictions.

**Variable**	**Type of vaccines**	***N***	**PRD-related reasons *N* (%)**	**No restrictions *N* (%)**
Missed vaccinations	Non-live	27	2 (7.4)	25 (92.6)
	Live-attenuated	21	18 (85.7)	3 (14.3)
Delayed vaccinations	Non-live	18	8 (44.4)	10 (55.6)
	Live-attenuated	12	6 (50.0)	6 (50.0)

PRD, pediatric rheumatic disease.

If vaccines for which there were no PRD-related restrictions had been administered, complete vaccination status would have been achieved in 85.9% of patients.

### Delayed mandatory vaccinations

In the study population of 156 patients, 1,230 mandatory vaccinations had been performed. 1,058 vaccinations (86.0%) were completed on time within the standard Slovak Immunization Schedule. Altogether 157 (12.8%) vaccinations in 61 patients (39.1%) were delayed. Due to incomplete documentation, it was not possible to analyze possible delays of 15 vaccinations (1.2%).

127 vaccinations (80.9% of all delayed vaccinations) were not performed on time before the onset of the PRD, mainly in the 1 year of life. 30 vaccinations (19.1%) were delayed during rheumatological follow-up (Table 3). The mean delay in patients already diagnosed with a PRD was 11 (SD = 13.4) and 15.9 (SD = 12.1) months for 18 non-live vaccinations and for 12 live-attenuated vaccinations, respectively. Considering disease activity and IST of these patients, 10 of 18 non-live and 6 of 12 live-attenuated delayed vaccinations could have been administrated 19 (SD = 12.7) and 6.5 (SD = 1.8) months earlier, respectively.

### Optional vaccinations

Optional vaccinations were administered in 61 patients (39.1%), most common additional vaccines were against rotavirus (*n* = 23), hepatitis A (*n* = 18) and pneumococcus (*n* = 18) (before the introduction of compulsory pneumococcal vaccination in Slovakia). The majority of optional vaccines had been administered before the onset of PRD. Only 14 patients (8.9%) were vaccinated with optional vaccinations during rheumatological follow-up (influenza *n* = 2, varicella *n* = 1, hepatitis A *n* = 2, *Human papillomavirus n* = 4, tick-borne encephalitis *n* = 5). Coverage rates for mandatory and optional vaccines are shown in Table 4.

**Table 4:** Coverage rates for mandatory and optional vaccines at the reference date.

**Mandatory vaccines**	***N* (%)**	**Optional vaccines**	***N* (%)**
Diphtheria, tetanus, pertussis	129 (82.7)	Rotavirus ^¥^	23 (28.9)
Poliomyelitis (IPV)	129 (82.7)	Hepatitis A	18 (11.5)
*Haemophilus influenzae type b*	154 (98.7)	Tick-borne encephalitis	11 (7.1)
Hepatitis B	154 (98.7)	Varicella	9 (5.8)
Pneumococcus	118 (75.6)^*^	*Human papillomavirus* (HPV)^¤^	4 (4.3)
Measles, mumps, rubella	133 (85.3)	Influenza	4 (2.6)
Complete vaccination status	117 (75.0)	Meningococcus	4 (2.6)

One prematurely-born child was immunized against respiratory syncytial virus.

* Including optional and mandatory vaccination (2009—introduction of compulsory pneumococcal vaccination in Slovakia).

¥ Calculated for 80 children born after 2010 when rotavirus vaccine became available in Slovakia.

¤ Calculated for 94 children 9 years of age and older at the reference date, since HPV vaccination is available (all four vaccinated were females, one with a diagnosis of jSLE).

### Pneumococcal vaccination coverage

We identified a group of 38 patients (24.4%) who had never been vaccinated against pneumococci. 35 individuals were not vaccinated because they were born before the introduction of mandatory vaccination against pneumococci in Slovakia (24). The remaining 3 patients were not vaccinated despite the established mandatory vaccination (personal belief *n* = 1, unstable epilepsy *n* = 1, unidentified reason *n* = 1). Pneumococcal vaccination rate significantly improved in patients born after the implementation of mandatory vaccination against pneumococci (96.8% vs. 43.6%, *p* < 0.001).

## Discussion

Increased susceptibility to severe infections in patients with PRDs (1, 2) and proven effectiveness (medical and economic) of vaccinations in the prevention of potentially serious infections (9) form the rationale for vaccinations in PRD patients. Despite the increased need for protection, vaccination coverage remains insufficient in this patient group (10–14), mainly due to concerns and limited evidence about safety and efficacy of vaccinations during IST (10, 17).

In our study, 75.0% of PRD patients were completely vaccinated, which is in line or slightly exceeds vaccination coverage found in similar studies (74% of 239 PRD patients from Switzerland, 64.7% of 187 PRD patients from Slovenia and 61% of 200 PRD patients from Canada) (10, 11, 13). Lower rates (49%) were achieved in 128 French children with auto-inflammatory diseases (12). However, in light of the long-standing history of mandatory vaccination in Slovakia with traditionally high vaccination rates (> 95% for all mandatory vaccines in the same year) (25), this result demonstrates the inadequacy of vaccination in our cohort of Slovak PRD patients.

Regarding specific vaccines, the highest coverage was found for vaccination against *Haemophilus influenzae type b* (Hib) and hepatitis B (HBV). This is explained by the Slovak Immunization Schedule (applicable to the entire cohort), as both vaccines are currently administrated only in the 1st year of life and thus before the onset of rheumatic disease in the vast majority of patients. Despite the high vaccine coverage against HBV, some studies have raised concerns about persistence of protective immunity after a period of IST, as has been documented, both, in patients with inflammatory bowel disease and PRD (26, 27). Hence, persistence of HBV seroprotectivity may have to be re-evaluated in adolescents with PRDs on IST (26, 27). Studies evaluating immunogenicity against Hib in PRD patients are not available.

Vaccination with live-attenuated vaccines has always been a matter of concern, reflecting the theoretical possibility of infection by attenuated vaccine strains, especially in patients on high-dose IST, including biologics (28). Hence, until recently, vaccination with live-attenuated vaccines was considered to be contraindicated during high-dose IST (18). This restriction still remains standard-of-care in patients on IST in general in many countries, including Slovakia.

According to current recommendations of the European League Against Rheumatism (EULAR) for children (2021) and for adults (2019) with rheumatic diseases (RD), live-attenuated vaccines are no longer contraindicated during IST and especially booster doses of MMR and varicella zoster virus (VZV) vaccination may be considered with caution during low-grade immunosuppression (19, 20). This is an important change, not only in the context of recent measles outbreaks in Europe and the United States, but also because of increased migration related to the war in Ukraine, as up to 75% of all measles cases in Europe were reported from Ukraine in 2019 (29–31).

Vaccine coverage with mandatory live-attenuated vaccines (MMR) in our cohort reached only 85.3%, similarly to other countries (10, 13). The lowest complete vaccination coverage was found in the group of 11–14 year old PRD patients, mainly due to missed MMR booster, and this age was found to be the strongest independent risk factor for incomplete vaccination status in our cohort. This can be plausibly explained by the standard Slovak Immunization Schedule that was implemented until the year 2019, and according to which, the MMR booster was administered at the age of 10–11 years. Due to recent measles epidemics in Slovakia and in Europe and decreasing immunogenicity in school children, the vaccination schedule in Slovakia had been changed in 2020 (the end of our data collection) to administer the MMR booster at the age of 4–5 years (32). Thus, with respect to the MMR booster, the original vaccination scheme can be considered unfavorable for PRD patients, as many of them already suffered from and were treated for their PRD with IST including bDMARDs, at the age of recommended MMR booster administration. The above change in the vaccination scheme may be one of the steps to improve MMR vaccination coverage in PRD patients in Slovakia, as the median age of disease onset in our cohort was 5 years and thus a significant proportion of patients will be vaccinated before the onset of PRD.

Surprisingly, vaccination rates for some non-live vaccines (diphtheria, tetanus, pertussis, polio) were similarly low or even slightly lower than rates for live-attenuated mandatory vaccines (82.7% vs. 85.3%, respectively). Analysis of PRD-related reasons showed the high proportion (92.6%) of possibly inadequate causes for missing non-live vaccines. We assume that several variables contribute to this problem. Most of them are not specific for non-live vaccines and represent a vaccination problem in PRD patients in general. Despite growing knowledge about vaccination in PRD patients, concerns about vaccination safety still remain among parents and also among physicians (10, 17). Vaccine hesitancy may be further exacerbated by the rise of anti-vaccination movements (33) and the unproven presumption, that immune-mediated diseases may be provoked by preceding vaccination (34).

The crucial tool for improving vaccination compliance is effective communication between healthcare professionals and parents (35), in particular between pediatric rheumatologists, primary care pediatricians and parents. A National Survey in Greece showed that 50% of primary care pediatrician were hesitant to adhere to the national vaccination scheme without the expert input of the pediatric rheumatologist (17). Targeted training of healthcare providers, systematization of work by creating an immunization algorithm, preliminary planning and sending reminders have proven to be effective measures in increasing vaccination rates against pneumococci in patients with PRDs (36). Also, a cross-sectional vaccination status assessment in our cohort has identified 10.9% of patients who could receive previously missed vaccinations, thus reaching an overall full vaccination status in 85.9% of PRD patients.

The overall vaccination coverage against pneumococci in our cohort was 75.6%. A lower rate was found among PRD patients in Slovenia (4.3%), where this vaccination is not mandatory, and a higher rate (87.1%) in Quebec, Canada, where pneumococcal vaccination has been part of the immunization program since 2004 (10, 13). Similarly, our data point to the effectiveness of the introduction of mandatory vaccination in improving the pneumococcal vaccination rate, as it increased from 43.6 to 96.8% when comparing patients born before and after the implementation of this public-health measure. On the other hand, identifying unvaccinated patients is always the first step in improving vaccination coverage. Based on international recommendations for PRD patients, we were able to plan this vaccination individually respecting the course of the underlying disease (19, 20).

In contrast to the traditionally high vaccination coverage with mandatory vaccines in Slovakia (>95% for all vaccines), the willingness to vaccinate with optional vaccines is generally very low. As an example, vaccination coverage against influenza reached only 4.2% for the entire Slovak population in the 2019/2020 season (25). Vaccination rates of Slovak children with influenza and other optional vaccines are not available. A similar study from Slovenia reported insufficient, but higher optional vaccination rates against influenza (10.2%), varicella (13.4%) and HPV (31.3%) in PRD patients (10). The most commonly administered optional vaccine in our cohort was against rotavirus, a live-attenuated vaccine administrated before the age of 6 months and thus before the usual onset of PRDs in our cohort. Vaccination rates against varicella (5.8%), influenza (2.6%) and meningococci (2.6%) are alarmingly low in our cohort, as these vaccinations are recommended for the majority of patients with RD, especially those on IST (19, 20, 23). Our data also point to low awareness of HPV vaccination in Slovakia, as only 4.3% of eligible children and only 1 child in 5 with jSLE had received HPV vaccination. Evidence of more frequent serious infections in patients with PRDs, especially respiratory tract infections, the higher risk of VZV complications, and more common anogenital complications of HPV infection in patients with jSLE (1, 2, 37, 38) warrant interventions to increase vaccination rates with these vaccinations in this vulnerable population.

Limitations of our study are related to the retrospective design and low response rate, as complete data were available from approximately half of the patients followed at our center. We cannot exclude, that parents who did not provide updated vaccination status information were less prone to comply with vaccination recommendations. On the other hand, data were accurate, as these were retrieved from primary care pediatricians and subsequently checked and updated to the reference date. As complete medical records of primary care pediatricians were not studied, reasons for missing mandatory vaccinations other than PRD may not have been fully appreciated and thus the impact of PRD on missing vaccination may be falsely overestimated. However, enquiries about infectious diseases and other medical conditions are standard of pediatric rheumatology care and thus are not likely to be missed. Additionally, implementation of electronic medical records, including a vaccination registry, in the future, may provide pediatric rheumatologist with more accurate data and thus enable more effective vaccination management.

## Conclusion

Our study points to insufficient vaccination coverage of PRD patients with mandatory vaccines, as only 75.0% of our cohort was fully vaccinated. Reasons for missing vaccinations unrelated to PRDs are frequent and possibly inadequate. There is a strong need to increase vaccination rate in this vulnerable group of patients. Immunosuppressive therapy had been placing restrictions on vaccination with live-attenuated vaccines until recently. In the light of current recommendations, this may not be adequate anymore and especially vaccination with MMR booster and VZV should be strongly considered also in the context of recent measles outbreaks all over the world as well as the potentially serious course of varicella in patients with systemic IST. Prospective collaborative efforts addressing the safety and efficacy of live-attenuated vaccines in PRD patients under immunosuppressive treatment are of utmost importance. Periodic vaccination status assessments, prospective planning of vaccinations and information interventions directed especially at groups identified to be at increased risk for incomplete vaccination status (biologic treatment and patients aged 11–14 years in Slovakia), may considerably promote full vaccination status in PRD patients and thus may be important measures in pediatric rheumatology care. These may be especially important also for optional vaccinations, as vaccination coverage with these vaccines is very low, and thus may require additional efforts to increase vaccination coverage.

## Data availability statement

The raw data supporting the conclusions of this article will be made available by the authors, without undue reservation.

## Ethics statement

The studies involving human participants were reviewed and approved by Ethics Committee of the National Institute for Children's Diseases, Bratislava, Slovakia. Written informed consent to participate in this study was provided by the participants' legal guardian/next of kin.

## Author contributions

BB and TD designed the study. ZK gave valuable advice on study design and manuscript writing. BB collected data, performed statistical analysis, and wrote the manuscript. TD, KN, and DM were responsible for patient care, data collection, and documentation handling. TD and ZK revised the manuscript. All authors contributed to the article and approved the submitted version.

## Funding

This study was supported by a grant from the NGO Zlatá rybka.

## Conflict of interest

The authors declare that the research was conducted in the absence of any commercial or financial relationships that could be construed as a potential conflict of interest.

## Publisher's note

All claims expressed in this article are solely those of the authors and do not necessarily represent those of their affiliated organizations, or those of the publisher, the editors and the reviewers. Any product that may be evaluated in this article, or claim that may be made by its manufacturer, is not guaranteed or endorsed by the publisher.
